# Parietal Encephalocele With Fenestrated Superior Sagittal Sinus and Persistent Falcine Sinus

**DOI:** 10.7759/cureus.16019

**Published:** 2021-06-29

**Authors:** Yekaterina Kokidko, Nathan Ranalli, Chetan Shah

**Affiliations:** 1 Pediatrics, University of Florida College of Medicine, Jacksonville, USA; 2 Pediatric Neurosurgery, University of Florida College of Medicine, Jacksonville, USA; 3 Pediatric Radiology, Nemours Children's Health System, Jacksonville, USA

**Keywords:** persistent falcine sinus, fenestrated superior sagittal sinus, parietal encephalocele, subependymal heterotopia, thick tectum

## Abstract

We present a case of a newborn with a fenestrated superior sagittal sinus and persistent falcine sinus with a parietal encephalocele. The patient was born full-term without any associated pregnancy complications other than meconium-stained amniotic fluid at delivery. Following delivery, MRI brain demonstrated midline parietal encephalocele, persistent falcine sinus, fenestration of the superior sagittal sinus at the level of the encephalocele, subependymal heterotopia, and thick tectum. The patient underwent resection and repair on day 2 of life. MRI performed at 15 weeks of life showed a mild increase in the size of lateral ventricles. The patient did not require a ventriculoperitoneal shunt. This is a novel case that provides a valuable contribution to the existing body of literature about congenital encephalocele associated with persistent falcine sinus, fenestrated superior sagittal sinus, subependymal heterotopia, and thick tectum.

## Introduction

An encephalocele is a congenital herniation of intracranial structures through a skull defect and is commonly associated with other intracranial anomalies. Because of the frequency of associated intracranial anomalies, publications focus on reporting novel anomalies found with encephaloceles. Prior publications report findings involving encephaloceles with isolated associated intracranial anomalies like elongation of the vein of Galen, persistent falcine sinus, fenestration of the superior sagittal sinus, corpus callosum agenesis, intracranial cysts, tentorial malformations, cerebellar vermis agenesis, hydrocephalus, and gray matter heterotopia [1,2]. However, reporting the rare concurrences of these anomalies could prove valuable to better understand them and contribute to existing literature.

## Case presentation

The patient is a newborn girl born at 40 weeks and six days of gestation via elective scheduled cesarean section. There were no pregnancy complications. Apgar scores at birth were 9 at 5 minutes and 9 at 10 minutes. Delivery complications included meconium-stained amniotic fluid. On physical examination at birth, the patient was noted to be awake and crying appropriately, moving all four extremities spontaneously without weakness, gross cranial neuropathies, or focal neurological deficits. Careful inspection of the calvarium revealed a soft and flat anterior fontanelle when the patient was calm and upright, head circumference was at the 31st percentile and a normocephalic configuration with the exception of a 3 x 3 cm soft, skin covered cephalocele at the midline of the posterior parietal scalp and skull overlying the posterior fontanelle or lambda. There was no evidence of drainage nor pulsatility and the associated bony defect approximated the circumference of the sac.

**Figure 1:**
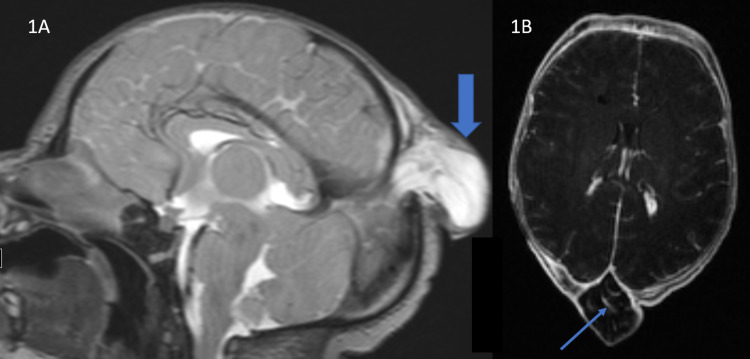
Parietal encephalocele T2-weighted sagittal MRI image (A) of the brain shows a bony defect in the midline with protrusion of dysplastic brain, CSF, and meninges through this defect consistent with parietal encephalocele (thick arrow). Persistent falcine sinus is present. Superior sagittal sinus is interrupted in the midline due to the encephalocele. T1-weighted axial post contrast MRI image (B) of the brain shows few enhancing vessels (thin arrow) in the dyspastic brain tissue within the parietal encephalocele.

**Figure 2:**
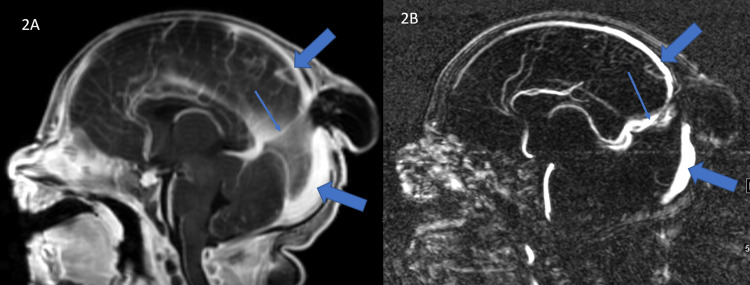
Persistent falcine sinus T1-weighted sagittal post contrast MRI image (A) and sagittal MR venogram image (B) show internal cerebral veins draining into the persistent falcine sinus (thin arrow) which drains into the superior sagittal sinus (thick arrow). The superior sagittal sinus is not seen in the midline at the site of encephalocele.

**Figure 3:**
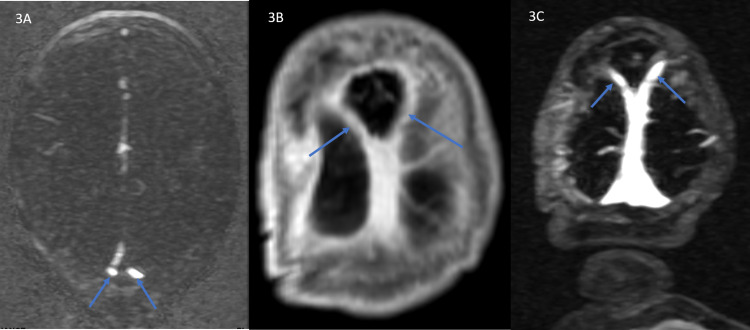
Fenestrated superior sagittal sinus Axial image (A) of magnetic resonance (MR) venogram of the brain at the level of encephalocele shows bifurcated (arrow) superior sagittal sinus. Coronal post-contrast T-1 weighted image (B) of the brain and MR venogram image (C) of the brain show the two limbs (arrows) of bifurcated superior sagittal sinus that reunites inferiorly to continue as an inferior part of the sagittal sinus.

Magnetic resonance (MR) imaging (MRI) and MR venogram (MRV) of the brain performed on day 1 of life demonstrated midline parietal encephalocele (Figure 1), persistent falcine sinus (Figure 2), and fenestration of the superior sagittal sinus (Figure 3) at the level of the encephalocele. The encephalocele contained dysplastic vascularized brain tissue covered by meninges. Encephalocele measured approximately 2.9 cm x 2.8 cm x 2.4 cm in cranial-caudal, ventral-dorsal, and transverse dimensions respectively. MR imaging of the brain also showed subependymal heterotopia and thick tectum. There was no hydrocephalus. MR imaging of the total spine was within normal limits.

**Figure 4:**
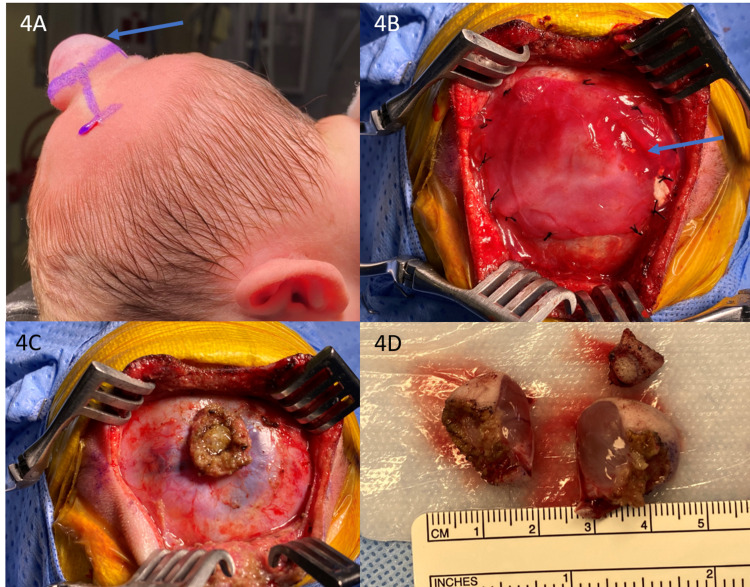
Surgical repair of encephalocele Photographs obtained during the surgery shows encephalocele (arrow) in the midline (A, B), appearance after resection of encephalocele (C) and surgical specimen (D).

The patient underwent resection (Figure 4) and repair of the posterior parietal encephalocele including duraplasty on day 2 of life. The MRI/MRV demonstrated a dysplastic brain tissue and fluid in the encephalocele fenestrating the underlying superior sagittal sinus at the posterior parietal vertex. The objective of the operation, therefore, was to circumferentially expose the defect in order to define normal surrounding dural elements, enter the sac and resect the dysplastic tissue down to the level of its pedicle at the site of the fenestrated superior sagittal sinus without violating the venous structure itself, and close the dura in a watertight manner. The repair was supplemented with a layer of dural allograft and the skin was brought together without significant tension. 

The patient tolerated the procedure without complication and she was monitored in the neonatal ICU for one week before being discharged home in good condition. Over the course of her hospital stay, she was followed closely for the potential development of hydrocephalus or cerebrospinal fluid (CSF) leak, neither of which occurred. The patient presented to our outpatient neurosurgical clinic several times over the next few weeks for routine wound assessment and management. Due to the development of superficial breakdown and scabbing at the surgical site, dedicated wound care nursing interventions were initiated. During her planned suture removal three weeks postop, a full-thickness dehiscence of the scalp was identified with exposure of the underlying dural allograft. The patient was subsequently taken to the operating room that same day for exploration and revision of her incision. No infection or CSF leak was found and she recovered well.

On postoperative day 22, the patient was noted to have evidence of full-thickness dehiscence of the scalp in the middle of the parieto-occipital incision with exposed underlying dural substitute. The patient was without signs or symptoms of infection or cerebrospinal fluid leak. However, given the full-thickness dehiscence and the low likelihood of healing without surgical repair, the wound was explored and revised in the operating room. There was no evidence of purulence or cerebrospinal fluid leak. The final wound culture was negative.

**Figure 5:**
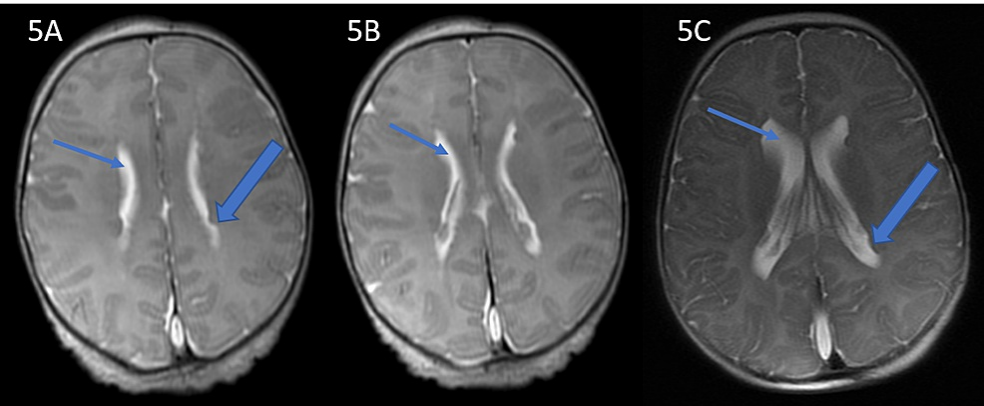
Ventricular size increase and heterotopia T2-weighted axial MRI of the brain on day 5 of life (A, B) and at 15 weeks of life (C) show interval mild increase in size of lateral ventricles (thin arrow) in addition to subependymal heterotopia (thick arrow).

**Figure 6:**
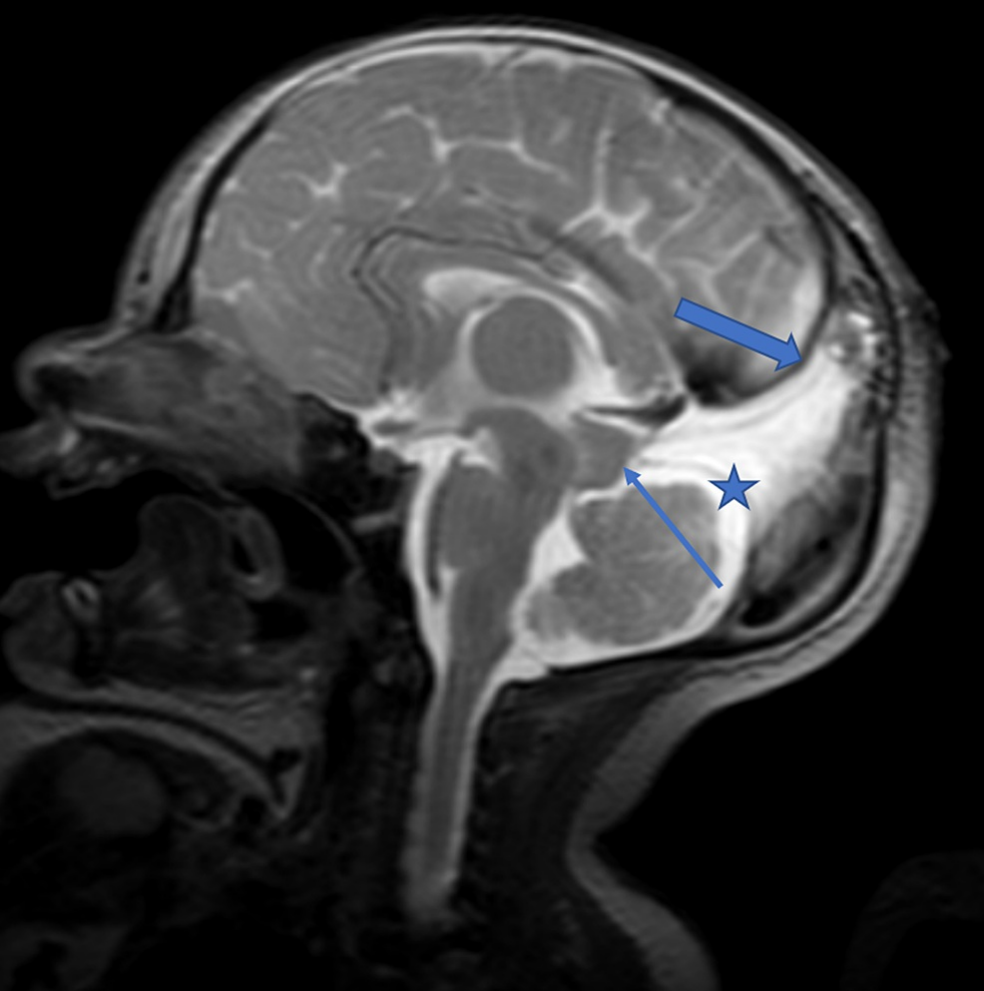
Thick tectum and increased CSF space surrounding the cerebellum T2-weighted sagittal MR image of the brain 15 weeks after the surgery shows the repair of encephalocele and presence of persistent falcine sinus (thick arrow), thick tectum (thin arrow), and excess CSF surrounding the cerebellum (star). CSF: cerebrospinal fluid

Her head circumference did increase in the days just after her encephalocele repair but a serial MRI scan (Figure 5) demonstrated subependymal heterotopia and mild enlargement of the ventricular and subarachnoid CSF spaces and she was without overt clinical or radiographic evidence of hydrocephalus. The patient was without any neurological symptoms or any focal neurological deficits on physical examination and did not require a ventriculoperitoneal shunt. MR imaging (Figure 6) of the brain performed at 15 weeks of life showed post-surgical changes of repair of the encephalocele and increased amount of CSF surrounding the cerebellum in addition to previously seen findings of persistent falcine sinus and thick tectum.

## Discussion

Cephaloceles are a congenital herniation of intracranial structures through a skull defect. If the lesion contains central nervous system (CNS) tissue, the term encephalocele is used and about half of encephalocele reported cases are in the parietal region [2]. Atretic cephaloceles are small, benign sub-scalp lesions that consist of meningeal and vestigial tissues without CNS tissue [3]. Commonly, encephaloceles are associated with other intracranial anomalies including elongation of the vein of Galen, persistent falcine sinus, fenestration of the sagittal sinus, corpus callosum agenesis, intracranial cysts, tentorial malformations, agenesis of the cerebellar vermis, hydrocephalus, and gray matter heterotopia [1,2].

Our case involves an encephalocele with multiple intracranial anomalies including a persistent falcine sinus in the presence of a fenestrated superior sagittal sinus, subependymal heterotopia, and thick tectum. The significance of the persistent falcine sinus and fenestrated superior sagittal sinus around the encephalocele is that if the surgeon attempts a more aggressive sac resection and/or exploration of the surrounding dura, then the underlying venous sinus may get damaged causing major bleeding which can be life-threatening in a newborn. Subependymal heterotopia may eventually cause seizures later requiring neurological consultation. Thick tectum may cause aqueduct obstruction and hydrocephalus. However, it appeared patent and yet the patient showed a mild increase in the size of the ventricles and increased CSF surrounding the cerebellum. Altered CSF hydrodynamics might explain some of these changes.

The falcine sinus is a typical fetus structure located in the falx cerebri, which connects the ventral part of the sagittal sinus and vein of Galen to the posterior one-third of the superior sagittal sinus [4]. The sinus normally involutes before or shortly after birth but is present in a small percentage of individuals after birth and is then known as a persistent falcine sinus. The persistence of the falcine sinus is thought to be a result of a mesenchymal disorder and may or may not be associated with other congenital intracranial anomalies. On the other hand, persistent falcine sinus can be acquired with recanalization after thrombosis or an obstruction of the straight sinus [4].

The sagittal sinus can appear as early as three months of gestational age and is a continuation of the primitive internal cerebral vein and median prosencephalic vein, the vein of Galen precursor [2]. Primordia of the superior sagittal sinus and transverse sinus appear as primitive marginal sinus around the 35th embryonal day. The left and right primitive marginal sinuses join at approximately the 50th embryonal day, followed by the formation of the superior sagittal sinus [5].

The treatment of encephaloceles is surgical. It is indicated to prevent the rupture of malformations, surgical management of a congenital encephalocele in the neonatal period is required to decrease the likelihood of sac rupture with associated CSF leak and risk for infection, including meningitis, and to eliminate the anomalous communication of intracranial contents with the extracranial space, allowing for normal brain and skull growth going forward. Surgical repair also helps relieve pain from possible dura stretch and also has a favorable cosmetic impact [1]. The approach and extent of resection are dependent not only on the contents within the sac, including the presence or absence of brain parenchyma, blood vessels, meninges, or CSF, but also on the location of the lesion. 

A review of the literature (Table 1) did not reveal any reported cases in the pediatric population of a parietal encephalocele containing neural tissue with both the findings of a persistent falcine sinus and fenestrated superior sagittal sinus in the same case. We reviewed the literature for other cases reported involving a cephalocele or encephalocele with intracranial anomalies that included either a persistent falcine sinus or a fenestrated superior sagittal sinus as these additional anomalies of dural sinuses can potentially pose a surgical challenge.

**Table 1:** Summary of associated anomalies in the literature

Year	Authors	Associated anomalies
1999	Otsubo et al	Fenestrated superior sagittal sinus
2009	Morioka et al	Fenestrated superior sagittal sinus
2010	Neumann J-O et al	Duplication of the superior sagittal sinus
2012	Hsu S-W et al	Persistent parietal falcine sinus and partial absence of the straight sinus
2014	Perez da Rosa et al	Persistent falcine sinus, bifid superior sagittal sinus, and absence of a transverse sinus, no neural tissue in cephalocele
2015	Siverino RO et al	Embryonic position of the straight sinus, fenestration of the superior sagittal sinus, abnormal insertion of the cerebellar tentorium with prominence of the superior cerebellar cistern and a septum pellucidum cyst
2021	Kokidko Y et al	Persistent falcine sinus, fenestrated superior sagittal sinus, neural tissue in cephalocele, subependymal heterotopia, thick tectum

Otsubo et al [5], reports a case with intraoperative findings that revealed a cephalocele penetrating a fenestrated superior sagittal sinus at the midline, but this case did not have a persistent falcine sinus. They also report several cases of parietal cephaloceles where the straight sinus is missing or not seen in the normal position. The straight sinus in our case, however, was present. Another case of a 19-month-old girl with fenestration of the superior sagittal sinus in association with a parietal cephalocele was reported by Morioka et al [6]. This case also had the presence of CSF in the parietal cephalocele just like in our case but septations or vessels were not appreciated in the reported case. A case of a 16-year-old female with a chief complaint of a midline parietal lump and worsening headaches who was found to have an atretic cephalocele with a persistent parietal falcine sinus and partial absence of the straight sinus but without a fenestrated superior sagittal sinus has been reported [2].

Additionally, a rare case of a duplication of the superior sagittal sinus with an associated parietal encephalocele has been published [7]. Following birth trauma, specifically vacuum extraction delivery, the patient had herniation and venous infarction of parietal brain tissue and the development of a subgaleal cerebrospinal fluid (CSF) collection. While our case did not involve any associated birth trauma, it is important to recognize the various complications that can derive early on in a patient’s life secondary to superimposed trauma on an existing encephalocele with associated intracranial anomalies.

Furthermore, a case of a three-year-old boy with an atretic cephalocele over the parietal region was associated with various cerebral anomalies of different midline structures including an embryonic position of the straight sinus, fenestration of the superior sagittal sinus, abnormal insertion of the cerebellar tentorium with the prominence of the superior cerebellar cistern and a septum pellucidum cyst. The cephalocele was composed of fibro-adipose tissue, dermal and meningeal elements without neural tissue or CSF as in the encephalocele in our case [3]. The atretic cephalocele was also not associated with a falcine sinus.

Moreover, a case most similar to our case is one reported by Perez da Rosa et al [8], in which a nine-month-old male presented with delayed growth and development, gait ataxia, and trouble swallowing. He was found to have an atretic cephalocele with a persistent falcine sinus, bifid superior sagittal sinus, and an absence of a transverse sinus. In this case, however, the atretic cephalocele did not contain any central nervous system tissue and there was no direct vascular connection between intracranial vessels and the extracranial mass. In our case, however, septations and vessels were present within the encephalocele which made the surgical procedure more complex and required more careful handling.

## Conclusions

In conclusion, we present this novel case of parietal encephalocele with multiple concurrent findings of persistent falcine sinus, presence of a fenestrated sagittal sinus, subependymal heterotopia and thick tectum. This case demonstrates the importance of evaluation of dural sinus anatomy that can pose a surgical challenge. The prognosis depends on the associated intracranial anomalies.
